# Editorial: Advances in Steroid-Responsive Encephalopathy

**DOI:** 10.3389/fneur.2020.00793

**Published:** 2020-08-06

**Authors:** Xin Tian, Xuefeng Wang, Patrick Kwan

**Affiliations:** ^1^Department of Neurology, The First Affiliated Hospital of Chongqing Medical University, Chongqing Key Laboratory of Neurology, Chongqing, China; ^2^Department of Neuroscience, The Central Clinical School, Monash University, Melbourne, VIC, Australia; ^3^Departments of Medicine and Neurology, University of Melbourne, Royal Melbourne Hospital, Parkville, VIC, Australia

**Keywords:** steroid-responsive encephalopathy, mechanism, diagnosis, treatment, prognosis

In neurology, “steroid-responsive encephalopathy” is a general term for diseases characterized by diffuse brain injury and responsiveness to steroids. These diseases include autoimmune encephalitis (AE), Hashimoto's encephalopathy (HE), limbic encephalitis, and anti-neutrophil cytoplasmic antibody (ANCA)-associated vacuities encephalopathy, among others. These diseases are common and complicated in clinical management. Further understanding of their epidemiology, pathophysiological mechanism, diagnosis, treatment and prognosis from various perspectives can help improve the insights of clinicians and researchers. To provide a platform for sharing the latest research findings in steroid-responsive encephalopathy, we organized this special issue, in which 11 manuscripts have been accepted for publication, including 6 original research articles, four reviews, and one mini review. To a certain extent, these manuscripts have expanded the current understanding of such diseases.

To date, there have been few large-scale epidemiological investigations of AE in adults or children, and its epidemiological characteristics remain unclear. Gu et al. provided a detailed description of the epidemiological characteristics of 189 patients with antibody-positive AE at six large general hospitals, and they also analyzed the differences in composition ratios, ICU occupancy, ventilator use, tumor and surgery, and prognosis among different age groups, gender groups, antibody groups, and disease characteristics. Separately, Qiu et al. retrospectively analyzed clinical features, laboratory and imaging results, and predictors of poor prognosis in 50 patients with an initial diagnosis of AE at their hospital. The authors found that the neutrophil-to-lymphocyte ratio might have predictive value for poor outcomes in AE and that early initiation of immunotherapy was associated with a good prognosis.

In a study focusing on pediatric AE, Zhang et al. retrospectively analyzed the clinical characteristics of 103 children with AE in two Chinese tertiary pediatric neurology centers, including 89 patients with anti-NMDA receptor (NMDAR) encephalitis, two with anti-LGI1 encephalitis, one with anti-CASPR2 encephalitis, and 11 autoantibody-negative patients with probable AE. Anti-NMDAR encephalitis is the most common form of AE in pediatric patients. Another study by Zhang et al. analyzed the demographic characteristics, clinical features, treatment, and outcomes of 34 children with anti-NMDAR encephalitis treated at Children's Hospital of Fudan University. The authors found that most of the included children were sensitive to first-line immunotherapy and achieved good outcomes, and higher Modified Rankin Scale scores before immunotherapy predicted poor outcomes. In addition, the authors also concluded that long-term use of antiepileptic drugs (AEDs) may not be necessary for pediatric patients with anti-NMDAR encephalitis.

Many patients with encephalitis have seizures during the development of the disease. Huang et al. followed up 75 outpatients with AE and reported in detail on the characteristics of those patients' seizures and their long-term use of AEDs. That study compared outcomes between patients with early and late AED withdrawal and determined the probable risk factors for seizure relapse and refractory epilepsy. As in Zhang et al. findings on pediatric anti-NMDAR encephalitis, these AE patients had a high rate of seizure remission after proper immunotherapy, and long-term use of AEDs may not be necessary to control their seizures. Compared with adults, young patients are more likely to become seizure free without AEDs. In addition, Huang et al. also reported that patients with anti-GABA_B_ receptor (GABA_B_R) antibodies, status epilepticus (SE), and cortical abnormalities had an increased risk of developing refractory epilepsy or seizure relapse.

A study by Lin et al. focused on the different clinical signs of infectious and autoimmune SE. These two entities may present with similar symptoms initially but require different treatment strategies. Since the prognosis of SE largely depended on etiology, faster-targeted treatment is required at the initial encounter. On this basis, Lin et al. conducted a retrospective study that included 501 patients with SE within a period of 10.5-years. Their study suggested that autoimmune SE had a relatively early age of onset; that it occurred predominantly in females; and that it often presented as psychosis, non-convulsive SE, and super-refractory SE. A lymphocytic predominance in cerebrospinal fluid was more commonly observed in patients with autoimmune SE than in those with infectious SE. These patient characteristics and signs may help clinicians select initial investigations and ensuing therapies that may improve overall outcomes.

HE has become increasingly recognized as an important and treatable cause of AE. Seizure disorders were observed in ~60–70% of patients with HE, and often as the first manifestation of the disease. HE is easily misdiagnosed because of the low incidence and the atypical symptoms. The manuscript by Li et al. discusses HE, the characteristic of its accompanying seizure disorders and the appropriate diagnostic approach.

Many neurologists may have limited experience in treating primary systemic vasculitis (PSV), mainly because most of these patients are diagnosed and managed by rheumatologists. However, PSV can affect every structure in both the central and the peripheral nervous systems, causing various neurological manifestations of dysfunction. Therefore, PSV patients may sometimes be referred to a neurologist first. The clinical manifestations of PSV are often non-specific, and differential diagnosis may be difficult. With these considerations in mind, Zhang et al. provide a comprehensive review of the clinical manifestations of PSV in the nervous system.

ANCA-associated vasculitis (AAV) is a multisystem inflammatory disease that can involve the central nervous system (CNS). Treatment with steroids, sometimes combined with immunosuppressants, can dramatically improve the outcome. However, for neurologists, the wide clinical spectrum of CNS involvement often complicates the diagnosis and thus delays treatment. Thus, Zheng et al. reviewed the manifestations of CNS involvement in AAV and emphasized ANCA testing, a crucial AAV diagnostic that requires appropriate result interpretation; the authors hoped to increase awareness and expand understanding of AAV-related CNS diseases among neurologists.

Immunoglobulin formulations have been used in an increasing number of diseases. In most cases, such formulations are safe and well-tolerated, but an increasing number of studies have reported potentially adverse effects of immunoglobulin treatment, some of which are severe and even fatal. In Guo et al. 's manuscript, the authors reviewed the incidence, risk factors and clinical characteristics of these adverse immunoglobulin-induced effects and addressed methods to minimize and prevent them.

Plasma exchange is widely used in the treatment of neurological diseases in which autoimmune mechanisms play a leading role. A growing body of research suggests that in the clinical treatment of steroid-responsive encephalopathies such as HE, limbic encephalitis, systemic lupus erythematosus encephalopathy, and ANCA-associated vacuities encephalopathy, plasma exchange is a safe and effective option when steroids or other immunosuppressive therapies are ineffective in the short term or when contraindications are present. A study by Jiang et al. provides a detailed review of the indications, onset time, course, curative effects, and side effects of plasmapheresis as applied clinically to steroid-responsive encephalopathy.

## Author Contributions

XT, XW, and PK organized this special issue and wrote the editorial. All authors have approved the final version of the editorial.

## Conflict of Interest

The authors declare that the research was conducted in the absence of any commercial or financial relationships that could be construed as a potential conflict of interest.

